# Dataset on the effect of perceived educational support on entrepreneurial intention among Vietnamese students

**DOI:** 10.1016/j.dib.2021.106761

**Published:** 2021-01-20

**Authors:** Hieu Thanh Nguyen, Doanh Cong Duong

**Affiliations:** National Economics University, Vietnam

**Keywords:** Perceived educational support, Attitude towards entrepreneurship, Subjective norms, Perceived behavioral control, Entrepreneurial self-efficacy, Entrepreneurial intention

## Abstract

This article illustrates the dataset that explores the effects of perceived educational supports on entrepreneurial self-efficacy, attitude towards entrepreneurship, subjective norms, perceived behavioral control and entrepreneurial intention. The scales from previous studies were adopted to develop the questionnaires using the five-Likert scale. 2218 fulfilled responses were included in the sample, which recruited from fourteen universities in Vietnam with the similar index. Also, a quantitative method was utilized to examine the dataset. Cronbach's alpha was used to test the reliability of each construct, then explore factory analysis was employed to estimate factor loadings of each observed variables and the validity and discrimination of variables was tested through confirmatory factor analysis. Then, the structural equation modelling was used to estimate the effects of perceived educational support on entrepreneurial intention as well as the other paths.

## Specifications Table

**Table:** 

Subject	Social Sciences
Specific subject area	Entrepreneurship
Type of data	Tables and figures and raw attached dataset
How data were acquired	Survey with the development of the questionnaire, which was modified from prior studies.
Data format	Raw data and analysed statistical data through SPSS 24.0 and AMOS 24.0 software
Parameters for data collection	Respondents who are final-year students at 14 universities/institutes in the Northern and Southern regions of Vietnam are voluntary to take part in the survey.
Description of data collection	Data was collected by stratified random sampling. The questionnaire was directly distributed to final-year students at 14 universities in Vietnam. The dataset included 2218 valid responses.
Data source location	City/Town/Region: 08 universities in the Northern region (National Economics University, National University of Civil Engineering, Hanoi University of Science and Technology, Dai Nam University, Foreign Trade University, University of Transport and Communications, Hanoi open university, Thuongmai University) and 06 universities in the Southern region (Quy Nhon University, Hue University, Da Nang University of Technology, Saigon University, University of Economics Ho Chi Minh City, Dong Thap University). Country: Vietnam Latitude and longitude for collected samples/data: 16.812913; 107.108177
Data accessibility	Data is included in this article.

## Value of the Data

• The data shows the perception of educational support and entrepreneurial intention among final-year students in 14 universities in Vietnam.
• The data reflects the correlations between perceived educational supports, attitude towards entrepreneurship, subjective norms, perceived behavioral control and entrepreneurial intention among Vietnamese students.
• The data can be served as useful reference sources for scholars who are interested in the entrepreneurship field.
• Exploring the effect of perceived educational supports on shaping entrepreneurial intention and discovering the mediating roles of attitude towards entrepreneurship, subjective norms, perceived behavioral control in the linkage between perceived educational support and entrepreneurial intention not only enables universities/institutes can build the appropriate educational environment to foster students’ business venture, but it also helps educational administrators and policymakers to develop the entrepreneurship ecosystem.

## 1 Data Description

Entrepreneurship field is interested in many scholars, educators, and policymakers because of its important roles in boosting economic growth, sustainable development of society and creating job opportunities. In the entrepreneurship field, the reasons why an individual intend or do not intend to create a new business venture has been interested in many scholars. Entrepreneurial intention is determined as a crucial concept in the early stage of the business venture process, a body of studies has concentrated on exploring its precursors. Thus, factors affecting the intention to start a business have also been explored by many researchers. For example, the influence of personal traits, entrepreneurial education, educational or gender difference on the entrepreneurial intention. However, the underlying mechanism of the relationship between perceived educational supports at universities and entrepreneurial intention is not clearly explained [1] although several recent studies also consider the relationships between university factors and entrepreneurial intention (e.g. Sieger & Monsen [2], Urbano et al. [3], Jena [4]). Indeed, the effect of perceived educational support on entrepreneurial intention is still inconsistent. Saeed et al. [5] and Turker & Selcuk [6] state that perceived education supports at universities are positively and strongly correlated with entrepreneurial intention while Soria-Barreto et al. [7] argue that the correlation between perceived university support and entrepreneurial intention is insignificant. Therefore, it has been suggested that this linkage should be further empirically explored [8]. Also, there are limited datasets of primary data which are available to investigate the influence of perceived education supports at universities on attitude towards entrepreneurship, subjective norms, perceived behavioral control, entrepreneurial self-efficacy and entrepreneurial intention among college students. As a result, it is necessary to have the primary dataset to discover these correlations; and most importantly, the data is essential to estimate the significant role of perceived educational support on shaping intention to engage in a business venture.

In addition, the raw dataset was utilized to illustrate the correlations between perceived educational supports, attitude towards entrepreneurship, subjective norms, perceived behavioral control and entrepreneurial intention. To specify, the dataset firstly aimed to provide the raw data, which was directly surveyed from college students, to show their perceptions of educational supports, attitude towards entrepreneurship, subjective norms, perceived behavioral control, entrepreneurial self-efficacy and entrepreneurial intention. Secondly, it aimed to show the statistical evidence of the important role of perceived educational supports in shaping entrepreneurial intention. Finally, the dataset presented the mediating role of attitude towards entrepreneurship, subjective norms, perceived behavioral control and entrepreneurial self-efficacy in the linkage between perceived educational supports and entrepreneurial intention.

To achieve these objectives, some measures from previous research has been adopted to develop the questionnaires. The survey administered to final-year students at 14 universities and institutes in Vietnam. The collected data was tested for validity and reliability, it was also employed to estimate the correlations of variables. Firstly, the Cronbach's Alpha, exploratory factor analysis (EFA), and confirmatory factor analysis (CFA) were utilized to test the validity and reliability of scales. Then, structural equation model (SEM) was used to estimate the correlation between variables.

Cronbach’ Alpha of all variables after extracting unsatisfactory items and exploratory factor analysis (EFA) were described in Table 1. The generally agreed upon lower limit for Cronbach's alpha is 0.70 [9]. Also, the value of the factor loading measured from latent variables via each observed variable (item) and reliability coefficient. Exploratory factor analysis (EFA) with Principal Axis Factoring (Promax) showed that KMO = 0.907, Sig. of Bartlett's Test of Sphericity = 0.000 < 0.005; Cumulative (%) = 72.226 > 50% and Eigenvalues = 1.016. Thus, the validity and reliability of all variables after extracting inappropriate observed variable were confirmed.

**Table 1:** Cronbach’ alpha and exploratory factor analysis after extracting unsatisfactory items.

	Factors					
Items	PBC	ATE	ESE	UE	SN	EI
α	0.822	0.826	0.840	0.846	0.851	0.918
PBC3	0.849					
PBC2	0.795					
PBC4	0.628					
PBC1	0.600					
ATE4		0.818				
ATE5		0.811				
ATE2		0.734				
ATE3		0.567				
ESE4			0.843			
ESE3			0.839			
ESE5			0.667			
ESE2			0.552			
UE1				0.862		
UE2				0.798		
UE3				0.754		
SN2					0.832	
SN3					0.793	
SN1					0.769	
EI5						0.847
EI4						0.839
EI6						0.803
EI3						0.790
KMO (Kaiser-Meyer-Olkin)			0.907			
Sig. (Bartlett's Test)			0.000			
Cumulative (%)			72.226			
Initial Eigenvalues			1.016			

*Note: N* *=* *2218; UE: Perceived educational supports at universities; ATE: Attitude towards entrepreneurship; ESE: Entrepreneurial self-efficacy; SN: Subjective norms; PBC: Perceived behavioural control; EI: Entrepreneurial intention; AVE: Average variance extracted; and CR: composite reliability.*

**Fig. 1:**
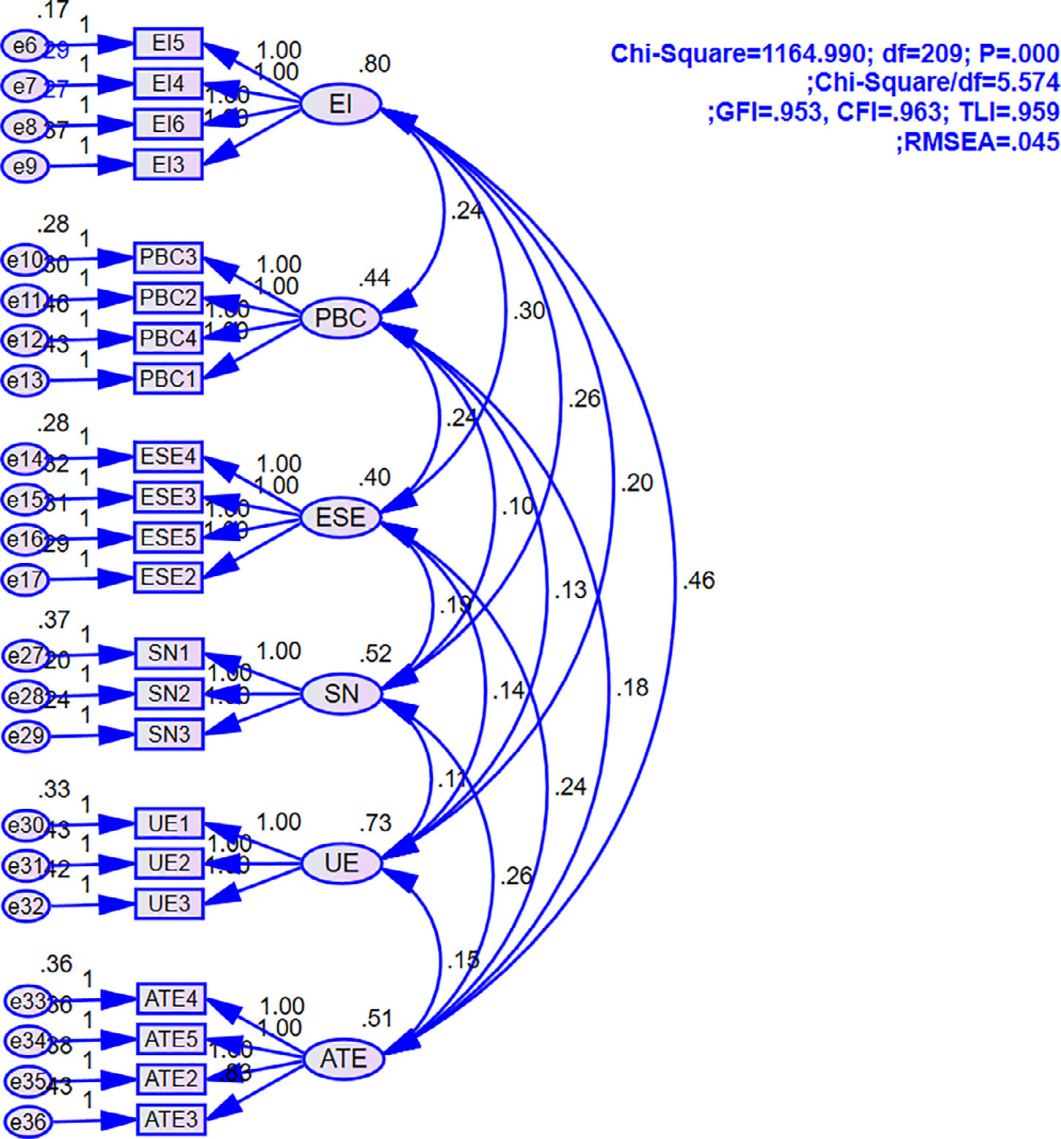
Confirmatory factor analysis.

Moreover, confirmatory factor analysis (CFA) was also employed to test the reliability, convergent and discriminant validity. The fit of model test results employing Chi-Square, CMIN/DF; CFI, GFI, TLI and RMSEA was summarized in Fig. 1. All mode fit indices match with recommended literature cut-off values when Chi-Square = 1164.990; Chi-Square/df = 5.574; GFI = 0.953; CFI =0.963; TLI =0.959; RMSEA = 0.045 [10].

Table 2 revealed the reliability, convergent validity as well as discriminant validity of each scales. CR values for all scales were higher than 0.7; AVE values of all scales were within their satisfactory level [11].

**Table 2:** The reliability, convergent and discriminant validity of each variable.

	CR	AVE	MSV	MaxR(H)	ATE	EI	SN	UE	PBC	ESE
ATE	0.829	0.549	0.523	0.833	**0.741**					
EI	0.922	0.746	0.523	0.926	0.723	**0.864**				
SN	0.855	0.663	0.261	0.860	0.511	0.398	**0.814**			
UE	0.848	0.650	0.070	0.849	0.245	0.257	0.179	**0.806**		
PBC	0.830	0.551	0.329	0.834	0.382	0.410	0.218	0.233	**0.742**	
ESE	0.840	0.567	0.329	0.840	0.540	0.533	0.427	0.264	0.574	**0.753**

*Note: N* *=* *2218; UE: Perceived educational supports at universities; ATE: Attitude towards entrepreneurship; ESE: Entrepreneurial self-efficacy; SN: Subjective norms; PBC: Perceived behavioural control; EI: Entrepreneurial intention; AVE: Average variance extracted; MSV: Maximum shared variance; and CR: composite reliability.*

**Fig. 2:**
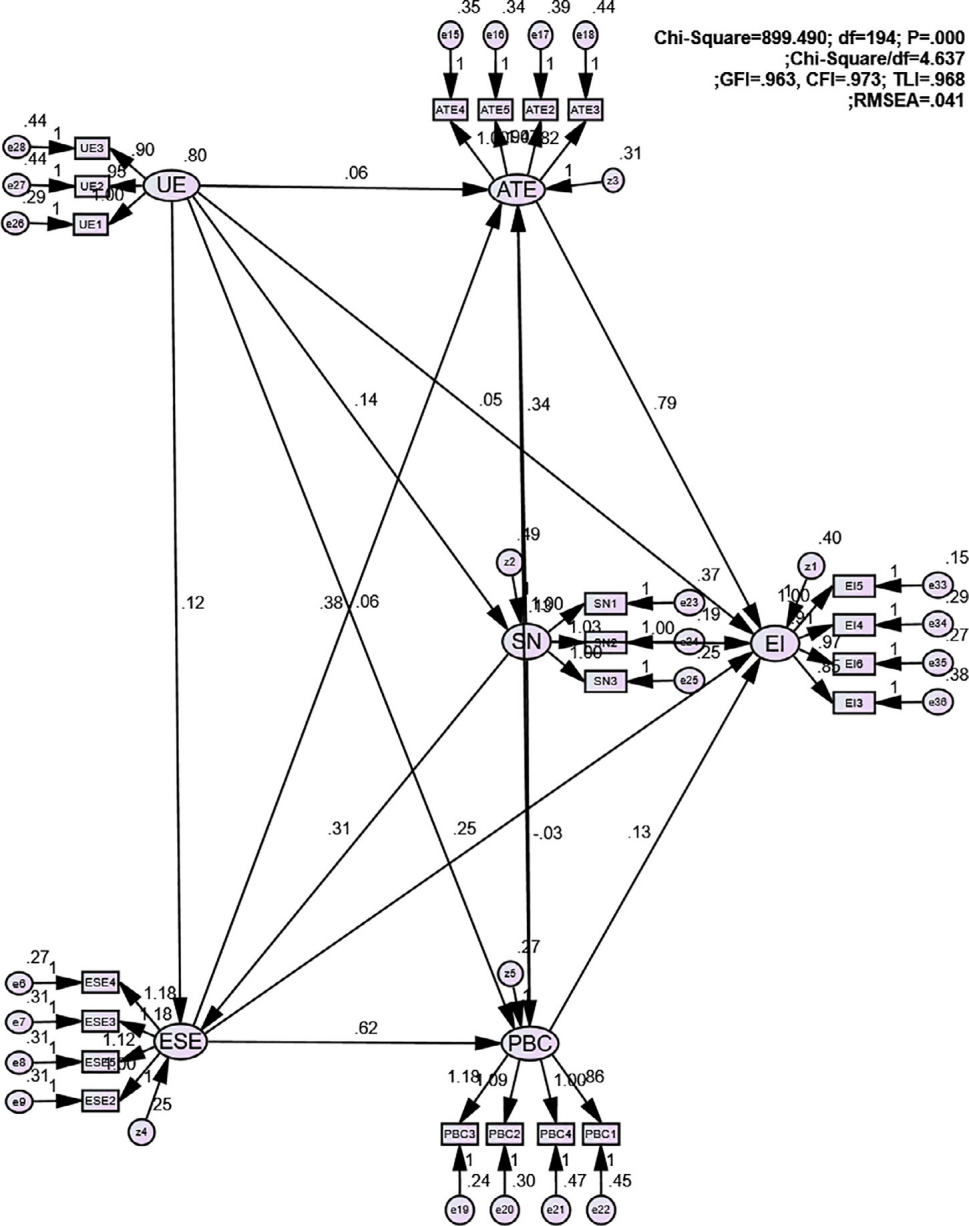
Path model.

The structural equational model (SEM) was utilized to estimate the linkages between variables. Fig. 2 represented that the model was completely fitted within the recommended levels: Chi-Square = 899.490; Chi-Square/df = 4.637; GFI = 0.963; CFI =0.9973; TLI =0.968; and RMSEA = 0.041.

Table 3 described the regression weights, which can be employed to estimate the relationship between statistical variables in the structural model. Structural equational model (SEM) analysis showed that entrepreneurial intention was directly correlated with attitude towards entrepreneurship (β = 0.786; p-value < 0.001), perceived behavioral control (β = 0.133; p-value < 0.001), entrepreneurial self-efficacy (β = 0.249; p-value < 0.001) and perceived educational support (β = 0.054; p-value = 0.006 < 0.01), but it was not affected by subjective norms (p-value > 0.005). Attitude toward entrepreneurship was directly affected by subjective norms (β = 0.343; p-value < 0.001), perceived behavioral control (β = 0.134; p-value < 0.001), entrepreneurial self-efficacy (β = 0.378; p-value < 0.001), and perceived educational support (β = 0.062; p-value < 0.001). Moreover, perceived behavioral control is direcltly related to entrepreneurial self-efficacy (β = 0.620; p-value < 0.001) and perceived educational control (β = 0.061; p-value < 0.001), but it was not involved in subjective norms (*p* > 0.05). Also, perceived education support was noticed to have direct effects on entrepreneurial self-efficacy (β = 0.062; p-value < 0.001) and subjective norms (β = 0.140; p-value < 0.001).

**Table 3:** Path coefficients and Regression Weights.

Paths			Estimates	S.E	C.R	P-value
Attitude towards entrepreneurship	→	Entrepreneurial intention	0.786	0.037	21.394	***
Subjective norms	→	Entrepreneurial intention	0.000	0.030	0.010	0.992
Perceived behavioral control	→	Entrepreneurial intention	0.133	0.035	3.774	***
Perceived behavioral control	→	Attitude towards entrepreneurship	0.134	0.031	4.281	***
Subjective norms	→	Attitude towards entrepreneurship	0.343	0.025	13.453	***
Subjective norms	→	Perceived behavioral control	−0.025	0.022	−1.143	0.253
Subjective norms	→	Entrepreneurial self-efficacy	0.307	0.020	15.027	***
Entrepreneurial self-efficacy	→	Attitude towards entrepreneurship	0.378	0.040	9.481	***
Entrepreneurial self-efficacy	→	Perceived behavioral control	0.620	0.035	17.611	***
Entrepreneurial self-efficacy	→	Entrepreneurial intention	0.249	0.046	5.464	***
Perceived educational support	→	Attitude towards entrepreneurship	0.062	0.018	3.501	***
Perceived educational support	→	Subjective norms	0.140	0.020	7.110	***
Perceived educational support	→	Perceived behavioral control	0.061	0.016	3.723	***
Perceived educational support	→	Entrepreneurial self-efficacy	0.121	0.015	7.999	***
Perceived educational support	→	Entrepreneurial intention	0.054	0.020	2.724	0.006

*Note: N* = 2218 ^⁎⁎⁎^ < 0.001.

Finally, 6000 bootstrapping samples with a confident degree of 90% was utilized to estimate indirect paths. Table 4 revealed direct, indirect, and total effects of factors on entrepreneurial intention among Vietnamese students. Bootstrapping analysis showed that perceived educational support had an indirect influence on entrepreneurial intention throughout entrepreneurial self-efficacy, perceived behavioral control, attitude towards entrepreneurship (β_indirect UE-EI_ = 0.214), but not via subjective norms. Subjective norms, although was not directly related to entrepreneurial intention, it indirectly affected entrepreneurial intention via attitude towards entrepreneurship and perceived behavioral control (β_indirect SN-EI_ = 0.477). Also, both perceived behavioral control and attitude towards entrepreneurship mediated the relationship between entrepreneurial self-efficacy and intention to create business ventures among students (β_indirect ESE-EI_ = 0.445). In addition, perceived behavioral control had indirect impact on entrepreneurial intention via attitude towards entrepreneurship (β_indirect PBC-EI_ = 0.106).

**Table 4:** Direct, indirect, and total effects.

		Independent variables				
Dependent variables	Effects	UE	SN	ESE	PBC	ATE
SN	Direct	0.140	0.000	0.000	0.000	0.000
	Indirect	0.000	0.000	0.000	0,000	0.000
	Total	0.140	0.000	0.000	0.000	0.000
ESE	Direct	0.121	0.307	0.000	0.000	0.000
	Indirect	0.043	0.000	0.000	0.000	0.000
	Total	0.164	0.307	0.000	0.000	0.000
PBC	Direct	0.061	0.000	0.620	0.000	0.000
	Indirect	0.098	0.190	0.000	0.000	0.000
	Total	0.159	0.190	0.602	0.000	0.000
ATE	Direct	0.062	0.343	0.378	0.134	0.000
	Indirect	0.132	0.138	0.083	0.000	0.000
	Total	0.194	0.481	0.461	0.134	0.000
EI	Direct	0.054	0.000	0.249	0.133	0.786
	Indirect	0.214	0.477	0.445	0.106	0.000
	Total	0.268	0.477	0.694	0.239	0.786

*Note: N* = 2218; UE: Perceived educational supports at universities; ATE: Attitude towards entrepreneurship; ESE: Entrepreneurial self-efficacy; SN: Subjective norms; PBC: Perceived behavioural control; EI: Entrepreneurial intention.

## 2 Experimental Design, Materials and Methods

**Fig. 3:**
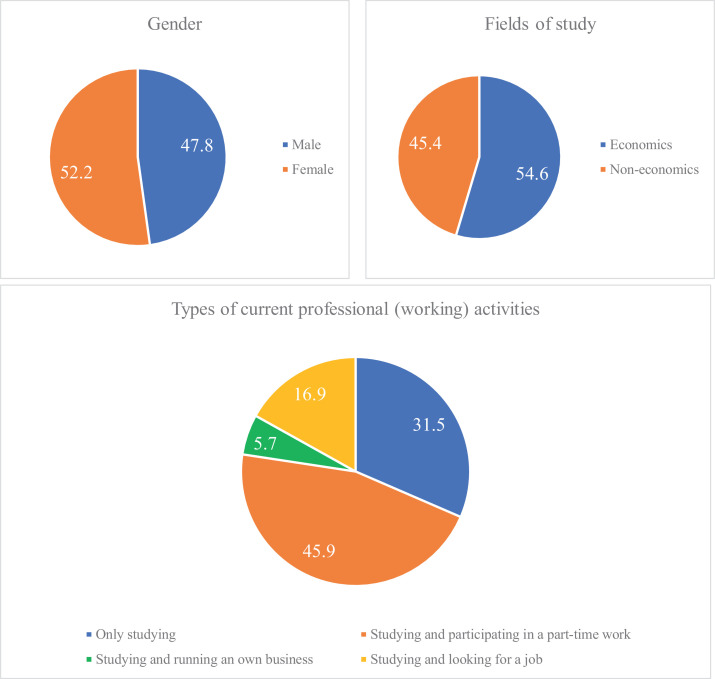
Sample characteristics (%).

The questionnaire has been adapted from previous studies [12], [13]. However, as the target respondents are students from Vietnam, therefore, the observed variables (items) were first translated into Vietnamese from the original English version. Some words have been modified to be more suitable to the Vietnamese context. Then, the questions were also back-translated into English to ensure the consistency between two versions. The questions were ranked in a five Likert-type scale from 1 (strongly disagree) to 5 (strongly agree). The scales were measured by five-point instead of seven-point Liker scale because many previous studies suggested that a five-point Likert scale should be utilized to increase response rate and quality with decreasing “frustration degree” of respondents [14] and improve reliabilities [15]. The questionnaire was delivered to students the first semester of the academic year 2018–2019. 2218 undergraduate students recruited from 14 universities in Vietnam has been included in the sample utilizing stratified random sampling with the four-phase procedure. At the first phase, two main regions of Vietnam (Northern and Southern areas) with the demarcation line in Quang Tri province, had been chosen. At the second sampling phase, the study chooses randomly eight universities in the Northern region (National Economics University (383 respondents), National University of Civil Engineering (73 respondents), Hanoi University of Science and Technology (198 respondents), Dai Nam University (42 respondents), Foreign Trade University (37 respondents), University of Transport and Communications (162 respondents), Hanoi open university (144 respondents), Thuongmai University (120 respondents)) and six universities in the Southern region (Quy Nhon University (212 respondents), Hue University (93 respondents), Da Nang University of Technology (181 respondents), Saigon University (148 respondents), University of Economics Ho Chi Minh City (209 respondents), Dong Thap University (216 respondents)). At the third stage, two to four classes each university were randomly selected. Then, research participants were recruited throughout the questionnaire that directly distributed to college students, who are final year students at these colleges/ universities. Students were clearly informed that they can participate in the survey voluntarily and their information will be secure and only use for the research purpose. The sample size and using stratified random sampling showed that the representativity of the sample was confirmed. Fig. 3 demonstrates the demographic characteristics of respondents.

This article utilized the quantitative approach to analyze the dataset and estimate correlation. Firstly, the Cronbach's alpha was used to examine the internal reliability of each construct, exploratory factor analysis (EFA) was also employed to consider factor loading of each item in variables, confirmatory factor analysis (CFA) was used to test the validity of each variable as well as the model fit. Then, structural equation modeling (SEM) was utilized to estimate the path coefficients. This was determined as an effective approach to examine the correlations. In addition, bootstrapping with a confident degree of 90% was utilized to show the indirect effects. Dataset was processed utilizing SPSS 24.0 and AMOS 24.0 software.

## Ethics Statement

The authors received informed consent from participants. Participants were voluntary, and they could withdraw from the survey at any point. As an ethical research team, we value the privacy rights of human subjects. Therefore, the data we submitted does not identify participants based on their responses. The survey was completely anonymous and does not contain any information allowing identifying the participants.

## CRediT Author Statement

**Duong Cong Doanh:** Conceptualization, methodology, data curation, investigation, writing-original draft preparation; **Nguyen Thanh Hieu:** Software, validation, reviewing and editing.

## Declaration of Competing Interest

The research project did not have financial supports or sponsors from any institutions/universities. The authors declare that they have no known competing financial interests or personal relationships which have, or could be perceived to have, influenced the work reported in this article.

## Appendix Supplementary materials

Image, application 1

Image, application 2

Image, application 3
