# Supplementary material for: Dataset on the effect of perceived educational support on entrepreneurial intention among Vietnamese students
Source: Data Brief. 2021 Jan 20;35:106761. doi: 10.1016/j.dib.2021.106761 (PMC7840465; doi:10.1016/j.dib.2021.106761)
Supplement: Supplementary file 1 [file mmc1.docx]

**QUESTIONNAIRE**

***Dear Students,***

*We are undertaking a research project on the influence of perceived educational supports on entrepreneurial intention among Vietnamese students. Please do me a favor by filling of this questionnaire. This questionnaire will be use for academic purpose only and will be analyzed and interpreted accounting to the principle of anonymity.*

*Thank you so much for your support!*

1. **ENTREPRENEURIAL INTENTION**

*Please answer the following questions with five-Likert scale:*

*1= Totally disagree*

*2= Disagree*

*3= Neutral*

*4= Agree*

*5 = Totally agree*

| Entrepreneurial intention | | | | | |
| --- | --- | --- | --- | --- | --- |
| 1. I am ready to do anything to be an entrepreneur | □1 | □2 | □3 | □4 | □5 |
| 2. My professional goal is to become an entrepreneur | □1 | □2 | □3 | □4 | □5 |
| 3. I will make every effort to start and run my own firm | □1 | □2 | □3 | □4 | □5 |
| 4. I am determined to create a firm in the future | □1 | □2 | □3 | □4 | □5 |
| 5. I have a very seriously through of starting a firm | □1 | □2 | □3 | □4 | □5 |
| 6. I have the firm intention to start a firm some day | □1 | □2 | □3 | □4 | □5 |

1. **FACTORS AFFECTING ENTREPRENEURIAL INTENTON**

*Please answer the following questions with five-Likert scale:*

*1= Totally disagree*

*2= Disagree*

*3= Neutral*

*4= Agree*

*5 = Totally agree*

| **Attitude towards entrepreneurship** | | | | | |  |
| --- | --- | --- | --- | --- | --- | --- |
| 7. Being an entrepreneur implies more advantages than disadvantages to me | □1 | □2 | □3 | □4 | □5 | |
| 8. A career as an entrepreneur is attractive for me | □1 | □2 | □3 | □4 | □5 | |
| 9. If I had the opportunity and resources, I’d like to start a firm | □1 | □2 | □3 | □4 | □5 | |
| 10. Being an entrepreneur would entail great satisfactions for me | □1 | □2 | □3 | □4 | □5 | |
| 11. Among various options, I would rather be an entrepreneur | □1 | □2 | □3 | □4 | □5 | |
| **Subjective norms** | | | | | |  |
| 12. If I decided to create a firm, my closest family would approve of that decision | □1 | □2 | □3 | □4 | □5 | |
| 13. If I decided to create a firm, my closest friends would approve of that decision | □1 | □2 | □3 | □4 | □5 | |
| 14. If I decided to create a firm, people who are important to me would approve of that decision | □1 | □2 | □3 | □4 | □5 | |
| **Perceived behavioral control** | | | | | |  |
| 15. To start a firm and keep it working would be easy for me | □1 | □2 | □3 | □4 | □5 | |
| 16. I am prepared to start a viable firm | □1 | □2 | □3 | □4 | □5 | |
| 17. I can control the creation process of a new firm | □1 | □2 | □3 | □4 | □5 | |
| 18. I know the necessary practical details to start a firm | □1 | □2 | □3 | □4 | □5 | |
| 19. I know how to develop an entrepreneurial project | □1 | □2 | □3 | □4 | □5 | |
| 20. If I tried to start a firm, I would have a high probability of succeeding | □1 | □2 | □3 | □4 | □5 | |
| **Entrepreneurial Self-Efficacy** | | | | | |  |
| 21. I show great aptitude for creativity and innovation | □1 | □2 | □3 | □4 | □5 | |
| 22. I show great aptitude for leadership and problem-solving | □1 | □2 | □3 | □4 | □5 | |
| 23. I can develop and maintain favorable relationships with potential investors | □1 | □2 | □3 | □4 | □5 | |
| 24. I can see new market opportunities for new products and services | □1 | □2 | □3 | □4 | □5 | |
| 25. I can develop a working environment that encourages people to try out something new | □1 | □2 | □3 | □4 | □5 | |
| **Perceived educational support** | | | | | |  |
| 26. The education in university encourages me to develop creative ideas for being an entrepreneur | □1 | □2 | □3 | □4 | □5 | |
| 27. My university provides the necessary knowledge about entrepreneurship | □1 | □2 | □3 | □4 | □5 | |
| 28. My university develops my entrepreneurial skills and ability | □1 | □2 | □3 | □4 | □5 | |

**C. PERSONAL INFORMATION**

1. Gender: □_1_ Male □_2_ Female
2. Field of studying:

□_1_ Economics

□_2_ Non-economics

1. University: ………………………………………………………………………
2. Current working and studying:

□_1_ Only studying

□_2_ Studying and participating in part-time job

□_3_ Studying and running an own business

□_4_ Studying and finding for a secure job
